# Supplementary material for: Factors associated with HIV viral suppression among adolescents in Kabale district, South Western Uganda
Source: PLoS One. 2022 Aug 18;17(8):e0270855. doi: 10.1371/journal.pone.0270855 (PMC9387807; doi:10.1371/journal.pone.0270855)
Supplement: Attachment — Submitted filename: Response to Reviewers.doc [file pone.0270855.s002.doc]

Siddappa N. Byrareddy, PhD
Academic Editor
PLOS ONE

Dear Byrareddy

We are writing to express our appreciation to you and the team of reviewers for carefully reviewing our manuscript titled “*Factors associated with HIV viral suppression among adolescents in Kabale district, southwestern Uganda’’ (pone-d-21-24368*) and to resubmit it for consideration of publication in PLOS ONE. The reviewers identified aspects of the manuscript that required revision. We are pleased to resubmit to you a revised version of the manuscript, which incorporates changes that have strengthened the overall manuscript. In the attached rebuttal memo, we describe how we addressed each of the points that the reviewers raised.

**Academic Editor;** *Please include your full ethics statement in the ‘Methods’ section of your manuscript file. In your statement, please include the full name of the IRB or ethics committee who approved or waived your study, as well as whether or not you obtained informed written or verbal consent. If consent was waived for your study, please include this information in your statement as well.*

**Response:** We thank the editor for identifying this gap. This has been addressed in the methods section. The study was approved by the Institutional Ethics Committee of Bishop Stuart University. Both verbal and informed written consent was obtained from adolescents aged 18 years and above while parents/legal guardians consented for the adolescents aged 10-17 years. The adolescents were also informed that their individual medical records would be reviewed and information extracted with outmost confidentiality.

**Reviewer #1;** *An abbreviation should be used after giving the expanded version of the same For e.g. HIV is abbreviated in the first line of background and then expanded in second line.*

**Response:** We thank the reviewer for identifying this error. We have reviewed and addressed the abbreviations.

**Reviewer #1;** Authors should review for grammatical errors.

**Reviewer #2;** There are many errors in English usage and the manuscript needs to be carefully proof read.

**Response:** We thank the reviewers for pointing out these grammatical errors. We have addressed the grammatical errors.

**Reviewer #2;** *The study lacks a central hypothesis. If the authors set out to sample the rate of viral load suppression in adolescent in the region, then the subject selection will need to be justified to represent the general HIV-1 infected adolescent population. The current subjects are skewed toward the 15-19 age group, which is unlikely to represent the general HIV-1 infected adolescent population in the region.*

**Response:** The study was conducted in Kabale district which is in south western Uganda. The word ‘’Kabale District’’ was missed in the title of the Manuscript. The study therefore did not assess viral load suppression in the whole south west region. The study subjects were selected from all the nine (9) sites providing adolescent HIV services in Kabale district and therefore the findings are representative of the district. At the time of the study a total of 363 adolescents were receiving HIV care in Kabale district. Sample size was estimated using a standard formula by (Kish & Leslie, 1965), with 5% marginal error and 95% confidence interval to arrive at a sample size of 239 participants which represented 65% of the total adolescents in HIV care. The sample size was then adjusted for missing data, and non-response; adjusted sample size was computed as 252 respondents. Probability proportionate to size sampling was used in this study because of the varying ART clinic sizes. This was aimed at ensuring that adolescents from larger ART clinics had the same probability of being included in the sample as those in smaller. The data was therefore not skewed towards the 15 -19 age group.

**Reviewer #2;** *The introduction section does not have key review of the field. At least ten similar studies have already been published and cited in the discussion section, which are related to viral suppression rates in HIV-1 infected adolescents.*

**Response:** We thank the reviewer for identifying this omission. We have revised this section to include the key review under the introduction section.

**Reviewer #2;** *Furthermore, the identified clinical and social factors associated with viral load suppression are largely expected. Therefore, it is unclear how the current manuscript will provide new information of interest to the scientific community.*

**Response**: We thank the reviewer for this important question. Although we acknowledge that the findings were largely expected, not many studies have been done to find out the HIV viral associated factors among adolescent especially in the rural settings and specifically in Kabale district. These findings will also provide age specific HIV viral load suppression rates which the routinely collected HIV program data does not provide and therefore will inform designing age specific interventions. The findings will also inform the Kabale district health department on their progress on the attainment of the 90:90:90 UNAIDS target.

**Reviewer #2;** *Part of written informed consent are not from parent/guardian but from care providers.*

**Response:** We thank the reviewer for identifying this. This was a wrong choice of word ‘’caregivers’’ informed consent was actually sought from the parents and guardians. We have made the corrections to use parents/guardians. e.g. ‘’ ……. parents/legal guardians consented for the adolescents aged 10-17 years’’.

**Reviewer #2;** *It is unclear how the key Odds Ratios statistical association were performed for the clinical and social factors. In Tables 4 and 5, percentages of many variables look close enough between viral suppression and suppression groups, yet many p values have reached the significant mark. With the modest sample size, it will be more meaningful to present data in dot pot association graphs rather than the current tables.*

**Response:** Thank you for pointing this out. It would have been interesting to explore this aspect of analysis. However, in the case of our study, we believe that it would be more appropriate to use logistic regression to generate the odds ratios at bivariate to see the relationship that exists between two variables, and multivariate to see if any, are correlated with the outcome variable than the dot plot. Furthermore, multivariate logistic regression model was used to estimate factors associated with viral load suppression adjusting for potential confounders. The multivariate logistic regression model employed a backward stepwise analysis to include all the variables in the model with p< 0.05 in selecting the final model. Considering our sample size, the authors think the dot plot will become overcrowded, making it difficult to read since the points may become cluttered. In addition, a dot plot is best when the sample size is less than approximately 50. The goal in this case was to determine which variables influence or cause the outcome.

**Reviewer #2;** *The authors is over-interpreting the association data. Psychosocial (or even general psychological factors) attributes have a correlated response to lower viral suppression because of either mismanagement of treatment or default of the regiment all together. However, the authors phrase it, making it sounds like a direct influence (as in a biological factor).*

**Response:** We thank the reviewer for this excellent suggestion. The authors think the psychosocial mechanisms may influence retention and adherence to treatment since issues like stress, traumatic events, and depression can lead to decreased CD4 counts and increased viral load, and therefore, accelerate HIV disease progression. In addition to the biological mechanisms that link HIV and psychological factors, social factors contribute to poor mental health outcomes among adolescents living with HIV. Because, the psychosocial problems can adversely affect adherence to ART and may lead to poor HIV treatment outcomes, the country opted for routine psychosocial factors screening as part of adherence support.

**Reviewer #2;** *Abstract section:  under Conclusion, they capitalized Viral when it doesn't need to be.*

**Response:** We thank the reviewer for pointing out this error. We have edited the sentence ‘’ …. findings indicate that HIV viral suppression among HIV positive adolescents ……’’

**Reviewer #2** *Background:  I'm not sure what they mean by "die owing."  Would be better to say, "die due to."  For a more technical sentence it would be more appropriate to say Adolescent's mortality is lower instead of the current vernacular.*

**Response:** We thank the reviewer for suggesting a more technical sentence. There is no doubt that use of technical terms will strengthen our paper. We have rephrased the sentences to read ‘’ The mortality of adolescents living with HIV due to acquired immune deficiency syndrome (AIDS)-related causes is higher than in adults (UNICEF, 2018)’’.

**Reviewer #2** *At the end of the Background section: age disaggregation groups is an odd choice in adjective conjugation.  More appropriate to say, "This is because age groups are disaggregated into adolescents between the ages of 10-15 years..."*

**Response:** We thank the reviewer for providing this guidance. We have edited the sentence to read;’’ This is because data for adolescent aged 10–15 years is lamped up under the age desegregation of 0–14 years’’

**Reviewer #2** *Last line of Sample Size and Sampling Procedures: HIV lived experiences just does not sound correct.  More appropriate to say, "...while adolescent peers shared their experience living with HIV."*

**Response:** We thank the reviewer for this correction. We have edited the sentence to read; …….’’ while adolescent peers shared their experience of living with HIV’’."

**Reviewer #2** *Under psychosocial factors, qualitative data: They used "lived experiences" again.  It's just an odd way of saying current life experiences dealing with HIV.*

**Response:** We again thank the reviewer for this correction. We have made the correction … ‘’while adolescent peers were interviewed to share their life experience dealing with HIV and taking lifelong ART’’.

**Reviewer #2** *Negative treatment from health workers section: This is clearly supposed to say, "...and they feel unwelcome at the health facility."*

**Response:** We thank the reviewer for pointing out this concern. We have revised the text for the sentence to read as follows; ….’’ Results further show that adolescents felt unwelcome at the health facilities because some of the health workers made adolescents uncomfortable in getting care and treatment.

Thank you for your time. We believe these revisions have resulted in a significantly improved manuscript.

We look forward to hearing from you in the due time regarding our submission and to respond to any further questions and comments you may have.

Sincerely,

Tugume Gordon Peterson.
