# Supplementary material for: Factors associated with HIV viral suppression among adolescents in Kabale district, South Western Uganda
Source: PLoS One. 2022 Aug 18;17(8):e0270855. doi: 10.1371/journal.pone.0270855 (PMC9387807; doi:10.1371/journal.pone.0270855)
Supplement: Attachment — Submitted filename: comments-PLoS ONE-1.docx [file pone.0270855.s003.docx]

The authors have addressed a majority of the concerns.

Second paragraph of Background section: “Two recent studies from southern Africa reported that HIV positive adolescents had worse outcomes in terms of virological suppression and rates of virological failure.”  **Worse** as compared to? Also the two **citations**?

Third paragraph of Result section. Data in this paragraph are from Table 4, but there is no reference of **Table 4** in the text.

Fourth paragraph of Result section: “**associated to**” should be “**associated with**”.

Qualitative findings section: “on the **on**”, please remove the second “**on**”.

Theme I: Support from significant others section: It is unclear why the authors discussed **significant others** when 98.8% of the subjects were single. Furthermore, there is no specific data from Tables 5 and 6 showing that having significant others increases viral load suppression.
