# Supplementary material for: Factors associated with HIV viral suppression among adolescents in Kabale district, South Western Uganda
Source: PLoS One. 2022 Aug 18;17(8):e0270855. doi: 10.1371/journal.pone.0270855 (PMC9387807; doi:10.1371/journal.pone.0270855)
Supplement: Attachment — Submitted filename: Response to Reviewers.doc [file pone.0270855.s004.doc]

Siddappa N. Byrareddy, PhD
Academic Editor
PLOS ONE

Dear Byrareddy

We are writing to express our appreciation to you and the team of reviewers for carefully reviewing our manuscript titled “*Factors associated with HIV viral suppression among adolescents in Kabale district, southwestern Uganda’’ (*PONE-D-21-24368R1) and to resubmit it for consideration of publication in PLOS ONE. The reviewers identified aspects of the manuscript that required revision. We are pleased to resubmit to you a revised version of the manuscript, which incorporates changes that have strengthened the overall manuscript. In the attached rebuttal memo, we describe how we addressed each of the points that the reviewer raised.

**Reviewer #2;** Second paragraph of Background section: “Two recent studies from southern Africa reported that HIV positive adolescents had worse outcomes in terms of virological suppression and rates of virological failure.”  **Worse** as compared to? Also the two **citations**?

**Response:** We thank the reviewer for pointing out this omission. We have removed this statement since there is no comparison that was stated.

**Reviewer #2;** Third paragraph of Result section. Data in this paragraph are from Table 4, but there is no reference of **Table 4** in the text.

**Response:** We thank the reviewer for identifying this omission. We have referenced table 4 in the text.

**Reviewer #2;** Fourth paragraph of Result section: “**associated to**” should be “**associated with**”.

**Response:** We thank the reviewer for this correction. We have edited the sentence to read; ……. “*associated with’’*…

**Reviewer #2;** Qualitative findings section: “on the **on**”, please remove the second “**on**”.

**Response:** We again thank the reviewer for this correction. We have made the correction and removed the second ‘’on’’ for the sentence to read ‘’…. findings on the psychosocial factors associated with….

**Reviewer #2;** Theme I: Support from significant others section: It is unclear why the authors discussed **significant others** when 98.8% of the subjects were single. Furthermore, there is no specific data from Tables 5 and 6 showing that having significant others increases viral load suppression.

**Response:** We thank the reviewer for raising this critical issue. We appreciate that most significant others are persons who are important to one's well-being especially spouses or one in a similar relationship. However, in this study we also included guardian, family members and others persons including peers who provided support to adolescent including accompanying them to the clinics and provided reminders to take medicine which was important in enhancing adherence to ART.

**Others**

We have also included a statement at the end of methods section on how qualitative data collected from Key informants was analysed.

Thank you for your time. We believe these revisions have resulted in a significantly improved manuscript.

Sincerely,

Tugume Gordon Peterson.
